# Acupuncture for patients with Maigne’s syndrome: A case series

**DOI:** 10.1097/MD.0000000000033999

**Published:** 2023-06-09

**Authors:** Hesol Lee, Hyocheong Chae, Myungseok Ryu, Changsop Yang, Sungha Kim

**Affiliations:** a Clinical Research Coordinating Team, Korea Institute of Oriental Medicine, Daejeon Republic of Korea; b Sunyujae Korean Medicine Clinic, Gyeonggi-do, Republic of Korea; c Daemyung Korean Medicine Clinic, Seoul, Republic of Korea; d KM Science Research Division, Korea Institute of Oriental Medicine, Daejeon, Republic of Korea.

**Keywords:** acupuncture, case series, Maigne’s syndrome, thoracolumbar junction syndrome

## Abstract

Diagnosing the precise etiology of low back pain (LBP) is crucial for facilitating speedy recovery in patients. Maigne's syndrome (MS), commonly referred to as thoracolumbar junction syndrome, is a condition characterized by pain resulting from nerve entrapment, yet its underlying mechanisms remain poorly understood. This study presents a series of six case reports wherein patients diagnosed with MS received acupuncture treatment.

**Patient concerns:**

Six individuals with LBP were included in the study, and all were diagnosed with MS.

**Diagnoses:**

The diagnosis was confirmed in all six patients through pinch-roll and thoracic vertebrae compression tests, indicating the presence of thoracolumbar junction syndrome.

**Interventions:**

Acupuncture treatment was administered to all patients, primarily targeting the T11–L2 facet joints, with additional acupoints selected based on the specific nerve entrapment of MS including the superior cluneal, subcostal, and iliohypogastric nerves.

**Outcomes:**

Following acupuncture therapy, all patients reported improvements in their LBP symptoms, while four patients also exhibited amelioration in their thoracic vertebrae compression test results.

**Lessons:**

These findings underscore the significance of promptly diagnosing the underlying cause of LBP and suggest that acupuncture may be an effective approach in alleviating MS-related pain.

## 1. Introduction

Low back pain (LBP) is one of the most prevalent types of pain affecting the human population. An initial episode of LBP occurs in 6.3% to 15.4% of the population, and the chance of recurrence within 1 year can be up to 90%.^[1]^ LBP is also ranked highest in terms of lived-with disabilities and ranked sixth in overall burden in terms of disability-adjusted life years, thus representing a real public health challenge.^[2]^ There is a wide variety of potential etiologies for LBP and one potential cause is Maigne’s syndrome (MS). Kuniya et al, in a prospective study of 834 consecutive patients in a surgical spine center clinic, reported the incidence of MS to be 14%.^[3]^

MS, also known as the thoracolumbar junction syndrome, is defined as pain caused by cluneal nerve entrapment, and pseudosciatica caused by the thoracolumbar lateral nerve branch.^[4,5]^ MS is poorly understood, and there are no specific clinical prediction rules or clear diagnostic criteria. This is because nerve-related pain is caused by soft tissue damage outside the spinal canal. Radiological findings are usually negative.^[6]^ A skin rolling test can also be performed to compare heaviness on either side.^[7]^ The superior cluneal nerve (SCN), subcostal nerve (SN), and iliohypogastric nerve (IN) have a direct connection with the thoracolumbar junction and the entrapment of those nerves are related to MS, commonly causing lumbosacral pain. If a patient responds sensitively while performing the pinch-roll test along the main distribution areas of the SCN, SN, and IN, the facet joint at the thoracic-lumbar junction should be palpated. The tenderness in the facet joint indicates a diagnosis of MS.^[8]^

Invasive treatments for MS include percutaneous rhizotomy, electrocoagulation, surgical denervation of the involved facet joint, superior and middle cluneal nerve release, and ultrasound-guided neural blocks.^[9–12]^ Conservative management approaches include a selective exercise regimen, corticosteroid or analgesic or anti-inflammatory medication, and spinal manipulative therapy.^[13–16]^ However, most of these treatments produce limited pain relief and are accompanied by serious side effects, such as drowsiness, dizziness, addiction, allergic responses, reversible reduction of liver function, and negative impacts on gastrointestinal functions.^[17–22]^ According to a Cochrane review, acupuncture is better than no treatment in terms of quick pain relief and functional improvement.^[23]^ Herein, we present 6 case reports with the aim of suggesting alternative treatments and provide insight into the effectiveness and safety of acupuncture for MS. This study was prepared in accordance with the CARE guidelines.^[24]^

## 2. Case presentation

### 2.1. Selection criteria of patients

**Figure 1.**
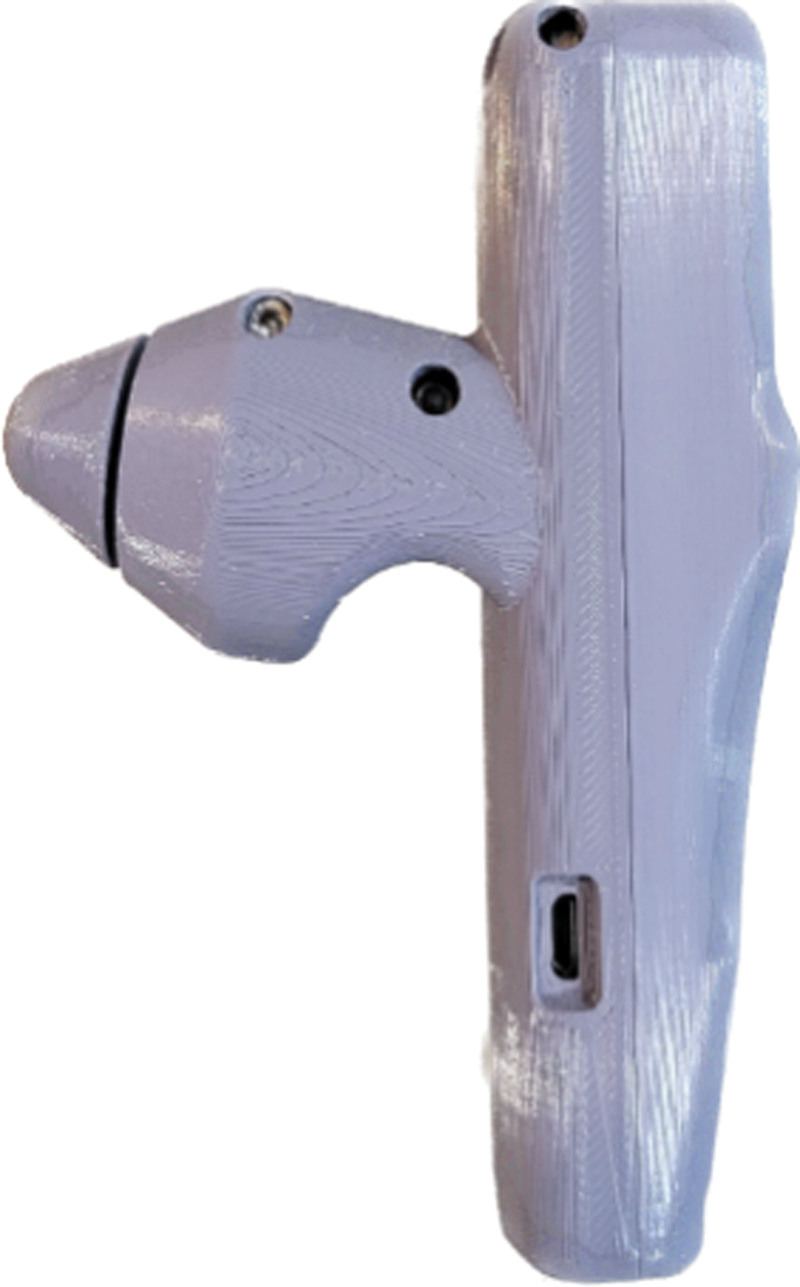
Weighing pressure sensor.

This This retrospective chart review was conducted at XX Clinic, and it included outpatients diagnosed with nonspecific low back pain (LBP) with MS who provided consent for publication, from January 1 to April 30, 2021. Patients with MS were diagnosed using the pinch-roll and thoracic vertebrae compression test (TVCT). The pinch-roll test was performed from the iliac crest on the back of the human body to the outside, which are the main distribution areas of the SCN, SN, and IN.^[25,26]^ The TVCT was applied to the facet joint areas of both T12 and L1, using combined modules: the Human Body Scale Sensor (Xin Nuo Qi Electric Co., Hong Kong, China) and HX711 Weighing Pressure Sensor (Hesai Technology, Palo Alto, CA). We pressed up to 83.36 N (8.5 kg kg•f) and measured the N that the patient feels tenderness (Fig. 1). Informed written consent was obtained from all patients. This study was approved by the Institutional Review Board of Korea institute of Oriental Medicine (I-2108/007-003).

### 2.2. Therapeutic intervention

#### 2.2.1. Acupuncture.

All patients with MS received acupuncture without variation. During the treatment period, 2 different acupuncture treatments were applied selectively, which were acupuncture or acupotomy. Overall, 8 to 10 acupoints were selected. The T11–L2 facet joint (Huatuo jiaji, Ex-B2 T 11-L2) were used identically in all patients, with 3 to 4 needles inserted at each pain side. We used 0.30 × 40 mm disposable stainless-steel needles (WOOJEON Acupuncture Needles, WOOJEON Co., Ltd., Seoul, Republic of Korea) with a depth of 3.0 to 4.0 mm, and the acupuncture time was 15 minutes.

**Figure 2.**
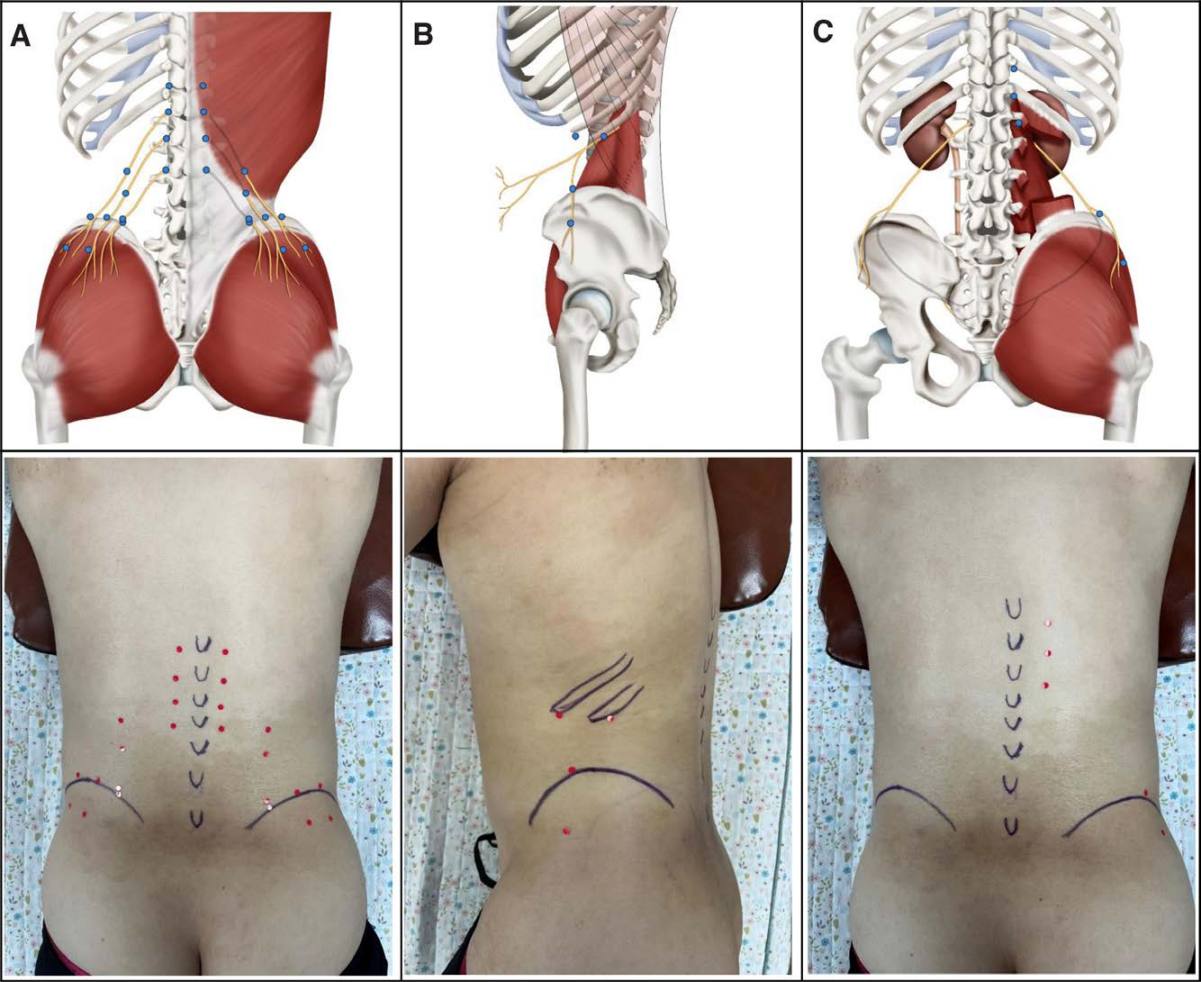
Acupoints of entrapment of nerves. (A) Acupoints of the superior cluneal nerve (B); acupoints of subcostal nerve; and (C) acupoints of the iliohypogastric nerve.

In case of entrapment of the SCN, where the SCN exits under the skin of the buttocks in the direction of travel of the SCN, 6 to 7 Ashi points were added, which were the tender points at the area of the nerve pathways (Fig. 2A). In case of entrapment of SN, 4 acupoints were added: 2 acupoints were inserted at the tip of the 11th and 12th ribs, tilting upward to the lower part of the costal tip, and 2 acupoints were inserted at the tender point where the SN exited under the skin of the buttock in the direction of the SN (Fig. 2B). In cases of entrapment of the IN, 2 to 3 acupoints were additionally inserted at the tender points where the IN exited under the buttock (Fig. 2C).

Acupotomy was applied when the patient’s disease period was over 3 months or other treatment like orthopedic manipulation did not work. We used 0.5 × 40 mm disposable stainless steel WOOJEON DOCHIM needles (WOOJEON Acupuncture Needles, WOOJEON Co., Ltd.) at the same acupoints as manual acupuncture.

#### 2.2.2. Other treatments.

Cupping was applied for 3 minutes. In cases of entrapment of the SCN, Huatuo jiaji of T12 and the tender points at the upper part of the iliac crest were used. In cases of entrapment of the SN, Huatuo jiaji of T12 and the tender points at the lateral iliac crest were targeted. In cases of entrapment of the IN, Huatuo jiaji of T12 and the tender points at the gluteus medius muscle were targeted.

Interference current therapy (ICT) was applied in all cases. In cases of bilateral pain (cases 1–3, 5, and 6), ICT was applied crossing the levator inferior and quadratus lumborum region. In the single case of unilateral pain (case 4), ICT was applied by crossing the levator inferior and quadratus lumborum upward and the gluteus medius downward.

A hot pack was applied from the lower part of the thoracic vertebrae to the lumbar sacrum.

Chuna was applied using a Leander Table (IWS-7000, Iwellness.co.kr, Korea). For the lumbar region and pelvis, the collateral lumbar distraction (lumbar roll), iliac posterior downward displacement correction, and anterior superior displacement correction techniques were used. For the thoracic region, the supine head flexion correction technique was used. After acupuncture, Chuna was performed once daily.

#### 2.2.3. Herbal medicine.

Hyeolbuchukeo-tang gami, which is most frequently used for cases of blood stasis in Korean medicine, shows anti-inflammatory and antinociceptive effects, and improves blood circulation, was used for cases when the period of disease was long.^[27]^ Table 1 lists the herbal medicines.

**Table 1:** Herbal medicines used in case 1 and case 6.

Case	Herbal medicine	Composition (per day)	Dose (period)
1, 6	Hyeolbuchukeo-tang gami	Bupleuri Radix 4 g, Cnidii Rhizoma 4 g, Rhei Radix et Rhizoma 4 g, Paeoniae Radix 4 g, Angelicae gigantis Radix 4 g, Trogopteronum feces 4 g, Akebiae caulis 4 g, Ponciri Fructus Immaturus 4 g, Carthami Flos 4 g, Achyranthis Radix 4 g, Lycopi Herba 4 g, Sappan Lignum 4 g, Rehmanniae Radix 4 g, Scutellariae Radix 4 g, Persicae Semen 4 g, Zingiberis Rhizoma Recens 4 g	2 packs (1 day)

### 2.3. Clinical outcomes

The Numerical Rating Scale (NRS), EuroQol-visual analogue scale (EQ-VAS), Roland-Morris Disability Questionnaire (RDQ), and TVCT were used. The NRS is used to measure pain, which rates pain intensity as an integer from 0 to 10.^[28]^ The EQ-VAS is a scale ranging from 0 to 100 (0 indicates the worst, 100 indicates the best condition), allowing the comprehensive judgment of one’s own health status.^[29]^ In addition, the RDQ is a patient-reported outcome (PRO) designed to assess physical disability due to LBP.^[30]^ The RDQ contains 24 items related specifically to physical functions likely to be affected by LBP derived from the Sickness Impact Profile, which is a 136-item health status measure covering all aspects of physical and mental function.^[31]^ Each question is limited to the phrase “because of my back pain.”^[32]^ The scores range from 0 (no disability) to 24 (maximum disability).^[33]^ The RDQ is better suited to patients for mild to moderate disability, and the Oswestry Disability Index to patients for persistent severe disability.^[33]^ Therefore, we used RDQ in association with outpatient treatment. Adverse reactions or side effects were confirmed through medical records. The TVCT was checked every time before acupuncture at both sides of the thoracic 12 (T12) and lumbar 1 (L1) vertebrae. In the TVCT, 83.36 N was used as a negative criterion. This is because, in our experience, when the patient did not feel pain even when the patient was compressed up to 83.36 N at entrapment of the SCN or SN or IN, pinch-roll tests, NRS, and lumbar flexion tests also showed negative results.

The characteristics of the cases are summarized and presented in Table 2 and patient-reported outcomes are summarized in Table 3.

**Table 2:** Characteristics of cases.

	Case 1	Case 2	Case 3	Case 4	Case 5	Case 6
Age, sex	45, Male	74, Female	55, Female	52, Male	54, Female	48, Male
Height (cm)/Weight (kg)	171/90	153/50	162/65	170/79	154/58	174/78
Chief complaint	LBP bilateral, both hips and inguinal area pain	LBP bilateral, belt distribution pain	LBP bilateral, pain when standing up to work	Left LBP and left flank pain	LBP, difficulty moving or turning	LBP bilateral, worse over the weekend
Diagnosis	MS, entrapment of SCN, SN, IN	MS, entrapped SCN	MS, entrapped SCN, IN	MS, entrapped SCN, IN	MS, entrapped SCN	MS, entrapped SCN, IN
Lumbar flexion test	+	+	+			
Tibial nerve compression test	−	ND	ND	ND		
Pinch-roll test	+, Region of SCNl,SN, IN	+, Region of SCN	+, Region of SCN & IN	+, Region of SCN & IN	+, Region of SCN	+, Region of SCN & IN
Thoracic vertebrae compression test		+	+	+	+	
Past history	Ureteral cancer, left kidney organ donation	−	Facial paralysis, cervical radiculopathy	−	−	−
Onset	2020-12-03	2021-04-25	2020-02-02	2021-02-24	2021-01-23	2021-03-13
Fist treatment date	2021-01-25	2021-04-26	2021-02-10	2021-02-27	2021-01-25	2021-03-19
Treatment period	3 times for 8 d	2 times for 3 d + 50 d (f/u)	2 times for 6 d+ 22 d (f/u)	3 times for 8 d	3 times for 8 d + 134 d (f/u)	2 times for 8 d
Interventions	Acupotomy, cupping, ICT, HP, HM	Acu, cupping, ICT, HP	Acu, cupping, ICT, HP	Acu, cupping, ICT, HP	Acu, cupping, ICT, HP	Acupotomy, cupping, ICT, HP, HM

Acu = acupuncture, f/u = follow up, HM = herbal medicine, HP = hot pack, ICT = interferential current therapy, IN = iliohypogastric nerve, LBP = low back pain, MS = Maigne’s syndrome, ND = no data, SCN = superior cluneal nerve, SN = subcostal nerve.

**Table 3:** Patient-reported outcomes of the 6 cases.

Date of test		Case 1		Case 2		Case 3		Case 4		Case 5		Case 6	
		Before treatment	After treatment	Before treatment	After treatment	Before treatment	After treatment	Before treatment	After treatment	Before treatment	After treatment	Before treatment	After treatment
		21.01.25	21.02.01	21.04.26	21.04.28.	21.02.10.	21.02.15.	21.02.27.	21.03.06.	21.01.25.	21.02.01.	21.03.19.	21.03.26.
NRS		5	1	5	4	6	3	7	3	4	2	5	1
EQ-VAS		30	65	40	48	15	30	40	50	20	40	5	35
RMDQ		10	6	12	11	2	0	11	7	4	3	10	6
Thoracic Vertebrae Compression test (N)	T12 (L)	57.07	71.98	43.64	57.96	62.37	67.08	79.34	69.33	44.13	57.47	Negative	Negative
	T12 (R)	66.88	Negative	49.33	50.90	56.88	53.45	Negative	Negative	Negative	Negative	Negative	Negative
	L1 (L)	55.70	71.49	55.51	Negative	62.08	60.02	78.65	72.28	49.33	60.51	66.19	Negative
	L1 (R)	76.10	Negative	56.09	Negative	74.82	63.74	Negative	Negative	Negative	Negative	62.86	74.73

EQ-VAS = EuroQol-visual analogue scale, L = left, N = Newton, NRS = numerical rating scale, R = right, RMDQ = Roland-Morris Disability Questionnaire.

#### 2.3.1. Case 1.

**Figure 3.**
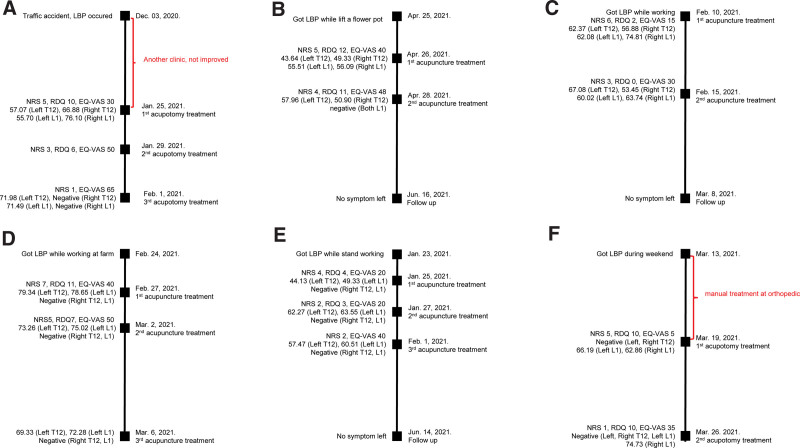
Time line of cases 1–6. (A) Timeline of case 1. (B) Timeline of case 2. (C) Timeline of case 3. (D) Timeline of case 4. (E) Timeline of case 5. (F) Timeline of case 6. EQ-VAS = EuroQol-visual analogue scale, LBP = low back pain, NRS = Numerical Rating Scale, RDQ = Roland-Morris Disability Questionnaire.

A 45-year-old male patient had a traffic accident on December 03, 2020. He was treated at another clinic and diagnosed with sprain in the lower back, but did not show any improvement after treatment. The first visit to our clinic was on January 25, 2021. At the first visit, the patient complained of bilateral LBP when standing, and the pain seemed to spread from the back to both hips and inguinal area. When he got up from a chair or the floor, his lower back did not straighten, and he experienced severe pain while turning when he layed down. The pain occurred during lumbar extension. He had a history of stage 3 ureteral cancer and a history of left kidney organ donation. As the tibial nerve compression test^[34]^ to rule out lumbar spinal canal stenosis and a pinch-roll test on the lower extremities were both negative, sciatic nerve entrapment and lateral femoral nerve syndrome were excluded. The pinch-roll test was positive in regions 1 and 2 (Fig. 2). This patient was diagnosed with MS with entrapment of SCN, SN, and IN. His NRS score was 5, RDQ score was 10, EQ-VAS was 30, and his TVCT result was 57.07 N for the left T12, 66.88 N for the right T12, 55.70 N for the left L1, and 76.10 N for the right L1. We treated 10 acupoints for the entrapment of SCNs, SN, and IN (Table 2). On January 29, 2021, the second visit, he was able to work to an extent, but the pain and difficulty remained when he got up after sitting. His NRS score was 3, RDQ score was 6, and EQ-VAS was 50. On the third visit, February 1, 2021, the pain almost disappeared: the left lower back area was slightly sore only when moving, and the right LBP had disappeared. His NRS score was 1, EQ-VAS was 65, and his TVCT was 71.98 N for the left T12, negative for the right T12, 71.49 N for the left L1, and negative for the right L1 (Table 3). No adverse effect was observed, except 2 local bruises occurred at the blood cupping sites, which disappeared soon. Figure 3A shows the timeline of case 1.

#### 2.3.2. Case 2.

A 74-year-old female patient developed acute back pain while lifting a flower pot on April 25, 2021. She arrived at the clinic on April 26 without visiting other clinics. She complained of pain in the form of a belt wrapped around the waist. Pain occurred during both lumbar flexion and extension. She had no radiating pain or abnormal sensations in the lower extremities. She was diagnosed with MS of SCN through the TVCT and pinch-roll test, and the acupoints were selected based on the diagnosis of MS with entrapment of SCN (Table 2). At the first visit, her NRS score was 5, RDQ score was 12, EQ-VAS was 40, and TVCT result was 43.64 N for the left T12, 49.33 N for the right T12, 55.51 N for the left L1, and 56.09 N for the right L1. At the second visit on April 28, 2021, the back pain had improved considerably. Her NRS score was 4, RDQ score was 11, EQ-VAS was 48, and TVCT result was 57.96 N for the left T12, 50.90 N for the right T12, and negative for the left and right L1 (Table 3). After the treatments, the pain disappeared. We followed-up with the patient on June 16th, and no pain was observed. Figure 3B shows the timeline of case 2.

#### 2.3.3. Case 3.

A 55-year-old slightly obese female patient with a history of facial paralysis and cervical radiculopathy, who not currently taking any medications, suddenly experienced LBP at work on February 10, 2021. She worked as an shop assistant. In particular, she suffered from back pain on both sides while standing at work, faced difficulty while standing for a long time, and had to rest. She did not have any radiating pain or any abnormal sensations in the lower extremities. She complained of severe pain during lumbar extension. She was diagnosed with MS syndrome of SCN and IN through the pinch-roll test and TVCT. She was treated with acupuncture on 10 acupoints based on the diagnosis of MS with the entrapment of SCN and IN on February 10 and February 15, 2021 (Table 2). On February 10, she complained of extreme pain when stretching her back, her NRS score scale was 6, RDQ score was 2, EQ-VAS was 15, and her TVCT result was 62.37 N for the left T12, 56.88 N for the right T12, 62.08 N for the left L1, and 74.82 N for the right L1. On February 15, 2021, she had improved a lot and the pain while stretching her back was also gone; her NRS score was 3, RDQ score was 0, EQ-VAS was 30, and her TVCT result was 67.08 N for the left T12, 53.45 N for the right T12, 60.02 N for the left L1, and 63.74 N for the right L1. When we followed-up with the patient on 8, March, 2021, the pain had disappeared. Figure 3C shows the timeline of case 3.

#### 2.3.4. Case 4.

With no past medical history, a 52-year-old male patient developed severe back pain while working at a farm on February 24, 2021. He mainly complained of pain in his left lower back and side, and the pain was severe enough to cause sleep disturbance. He visited this clinic on February 27, 2021 without visiting another clinic beforehand. MS with entrapment of SCN and IN was diagnosed through the pinch-roll and TVCT (Table 2). Ten acupoints were selected based on the diagnosis of MS with entrapment of the 2 nerves: SCN and IN.

His NRS score was 7, RDQ score was 11, EQ-VAS was 40, and his TVCT result was 79.34 N for the left T12, negative for the right T12, 78.65 N for the left L1, and negative for the right L1. The severe pain was slightly improved by the second visit on March 2, 2021, but he complained of a pulling symptom in his left flank. His NRS score was 5, RDQ score was 7, EQ-VAS was 50, and his TVCT result was 73.26 N for the left T12, negative for the right T12, 75.02 N for the left L1, and negative for the right L1. At the third visit on March 6, 2021, his NRS was 3 and his TVCT result was 69.33 N for the left T12, negative for the right T12, 72.28 N for the left L1, and negative for the right L1 (Table 3). The left LBP reduced. This patient tends to visit the clinic with frequent recurrence of similar symptoms because of doing repeated farm work every weekend. Figure 3D shows the timeline of case 4.

#### 2.3.5. Case 5.

A slightly obese 54-year-old female patient without previous diseases sprained her back on January 23, 2021 and visited the clinic on January 25, 2021 without visiting other clinics. On January 25, she complained of severe pain that made it difficult to move and to turn around. She was diagnosed with MS with the entrapment of SCN through the pinch-roll and TVCT (Table 2). Treatment was performed on 8 acupoints based on the diagnosis of MS with the entrapment of SCN. Her NRS score was 4, RDQ score was 4, EQ-VAS was 20, and her TVCT result was 44.13 N for the left T12, negative for the right T12, 49.33 N for the left L1, and negative for the right L1. The severe pain had improved a lot at the time of the second visit on January 27, and she could turn around and move more comfortably. Her NRS score was 2, RDQ score was 3, EQ-VAS was 20, and her TVCT result was 62.27 N for the left T12, negative for the right T12, 63.55 N for the left L1, and negative for the right L1. At the time of the third visit on February 1, the state of improvement was maintained similar to that of the second treatment. Her NRS score was 2, EQ-VAS was 40, and her TVCT result was 57.47 N for the left T12, negative for the right T12, 60.51 N for the left L1, and negative for the right L1 (Table 3). After the 3rd treatment, the pain disappeared. Figure 3E shows the timeline of case 5.

#### 2.3.6. Case 6.

A 48-year-old male patient got LBP during his weekend rest on March 13, 2021. He received manual treatment from an orthopedic before March 19, 2021, but the pain did not improve. He described bilateral back pain. MS with the entrapment of the SCN and IN was diagnosed through the pinch-roll and TVCT. Twenty acupoints were selected based on the entrapment of both side of SCN and IN (Table 2). When he visited the clinic on March 19, he had severe bilateral back pain; his NRS score was 5, RDQ score was 10, EQ-VAS was 5, and his TVCT result was negative for the left and right T12, 66.19 N for the left L1, and 62.86 N for the right L1.

On March 26, his pain improved markedly and he had almost no discomfort in his daily life; his NRS score at this point was 1, RDQ score was 6, EQ-VAS was 35, and his TVCT result was negative for the left and right T12 as well as the left L1, and 74.73 N for the right L1 (Table 3).

This patient was treated with acupotomy, as the pain was stubborn even when receiving orthopedic manipulation. Figure 3F shows the timeline of case 6.

## 3. Discussion and conclusion

This case series identified improvements in LBP due to MS through the use of acupuncture. The NRS, indicating pain, dropped to a value of 3 or less in all cases; scores in the EQ-VAS and RDQ, which measure the physical disability of patients with LBP, also improved. Interestingly, intractable LBP not responding to orthopedic manipulation was improved with acupuncture, with the understanding of the trigger points at the entrapment site of MS.^[12]^

MS can be misdiagnosed as pain is rarely felt at the thoracolumbar region, but rather presents in a similar fashion to LBP of the lumbosacral origin.^[35]^ Misdiagnosis of LBP due to MS leads to delayed appropriate treatments, and extends the treatment period.^[4]^ The main difference in LBP due to MS with that of herniated disc is the region of pain. Pain in patients with MS appears in the lower back and gluteal region, and radiating pain does not appear below the knee.^[36]^ We speculate that this is owing to the etiology of entrapment of the SCN, SN and IN in MS. This study was performed at a primary clinic, thus the skin rolling test and thoracic vertebrae compression test are most reliable tests to discern LBP due to MS from others like simple strain or sprain.

Isu et al^[12]^ reported on the surgical outcomes of SCN entrapment over 20 articles. However, only few studies on acupuncture for MS have been reported. Nevertheless, acupuncture has been used to stimulate myofasia.^[37]^ Here, in this study, we used acupuncture to stimulate myofasia, which caused the entrapment of nerves in MS. Acupotomy is a thicker acupuncture that make strong effect, and the treatment time is relatively short due to no need for retention.^[38]^ In addition, it recreates the original physiological state by facilitating the blood circulation of the tissue by exfoliating the adherent tissue.^[39]^ In Kim’s study, patients with acute LBP with NRS over 5 showed statistically significant NRS score reductions, improvements in the range of movement and RMDQ, improvements in EQ-5D and EQ-VAS scores, and satisfaction after acupotomy.^[40]^

Our study had strengths in that pain due to entrapment of nerves was improved easily with acupunture. Maigne claimed that articular facet syndrome with thoracolumbar junction syndrome and entrapment of the nerve at the iliac crest can be associated, requiring treatment of the spine and of the iliac crest.^[5]^ Following what Maigne claimed, we clinically identified the nerve entrapment points, which are the tender points as the “Ashi-point” – in which areas SCN, SN, and IN well captured. These Ashi-points are the specific treatment points, and the effect confirmed it as a target.

This study had the following limitations. Clinically, chronic MS is considered to recur frequently when there is a structural displacement of the spine, such as a compression fracture.^[41]^ This will also require a follow-up study to prove the correlation with the frequency of MS through structural evaluation of the spine. And due to the nature of the Korean primary medical clinic, treatments such as cupping, herbal medicine, and chuna were performed in parallel with acupuncture treatment. Thus, lack of control over multiple interventions is a limitation in confirming the effectiveness of acupuncture treatment through this study.

This study was considered to be a case series in which the characteristics of MS were effectively discriminated against patients with LBP in a primary clinic, and the treatment effect was quick. It is also significant in that this study is the first case series report of patients with MS who improved through a combination of Korean medicine treatments.

## Acknowledgments

We extend our sincere thanks to Hongmin Chu, Joohyun Lee for their contributions to this paper, including the figures.

## Author contributions

Conceptualization: Hyocheong Chae, Myungseok Ryu.

Funding acquisition: Changsop Yang.

Investigation: Hyocheong Chae, Myungseok Ryu.

Project administration: Changsop Yang.

Supervision: Sungha Kim.

Writing – original draft: Hyocheong Chae, Hesol Lee.

Writing – review & editing: Hesol Lee, Sungha Kim.
